# Self-stratified and self-powered micro-supercapacitor integrated into a microbial fuel cell operating in human urine

**DOI:** 10.1016/j.electacta.2019.03.194

**Published:** 2019-06-01

**Authors:** Carlo Santoro, Xavier Alexis Walter, Francesca Soavi, John Greenman, Ioannis Ieropoulos

**Affiliations:** a Bristol BioEnergy Centre, Bristol Robotics Laboratory, T-Block, UWE, Coldharbour Lane, Bristol, BS16 1QY, UK; b Department of Chemistry “Giacomo Ciamician”, Alma Mater Studiorum, Università di Bologna, Via Selmi, 2, 40126, Bologna, Italy; c Biological, Biomedical and Analytical Sciences, UWE, Coldharbour Lane, Bristol, BS16 1QY, UK

**Keywords:** Microbial fuel cell, Supercapacitor, High power density, Urine, Discharge, Self-powered

## Abstract

A self-stratified microbial fuel cell fed with human urine with a total internal volume of 0.55 ml was investigated as an internal supercapacitor, for the first time. The internal self-stratification allowed the development of two zones within the cell volume. The oxidation reaction occurred on the bottom electrode (anode) and the reduction reaction on the top electrode (cathode). The electrodes were discharged galvanostatically at different currents and the two electrodes were able to recover their initial voltage value due to their red-ox reactions. Anode and cathode apparent capacitance was increased after introducing high surface area activated carbon embedded within the electrodes. Peak power produced was 1.20 ± 0.04 mW (2.19 ± 0.06 mW ml^−1^) for a pulse time of 0.01 s that decreased to 0.65 ± 0.02 mW (1.18 ± 0.04 mW ml^−1^) for longer pulse periods (5 s). Durability tests were conducted over 44 h with ≈2600 discharge/recharge cycles. In this relatively long-term test, the equivalent series resistance increased only by 10% and the apparent capacitance decreased by 18%.

## Highlights

• Supercapacitive microbial fuel cells fed with human urine were investigated.
• Galvanostatic discharges were studied and key parameters presented.
• ESR and capacitance were improved by adding supercapacitive features.
• Highest power obtained was 1.20 ± 0.04 mW (2.19 ± 0.06 mW ml^−1^) for a t_pulse_ of 0.01 s.
• Stability over 2600 cycles of discharge/self-recharge (44 h) was shown.

## Graphical abstract

**Figure:**
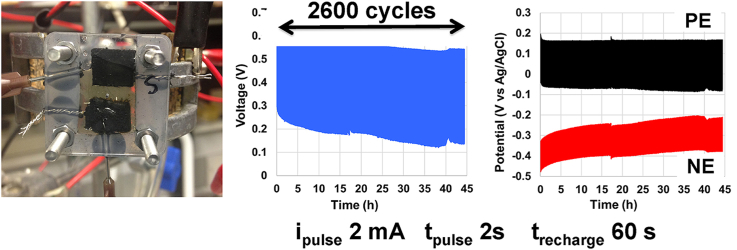

## 1 Introduction

Energy and water availability are two major problems that humankind will face in the next decades [[1], [2], [3]]. Recently, a completely new field named water-energy nexus was created with the purpose of optimising the existing technologies and reducing the consumption of water and energy for a more sustainable world [4,5]. For example, water is related to power for cooling, minerals mining, fuel production, emission controls, etc. [6]. In parallel, power is consumed for cleaning water, pumping, aeration, final distribution [7,8].

Bioelectrochemical systems (BES) are among the most interesting water-energy nexus technologies because of their capability to treat pollutants, clean the water and also produce electricity simultaneously or valuable added products (VAP) [[9], [10], [11], [12]]. The main idea is to use the wastewater or the waste product as a fuel for the system [13,14]. The common point among all the BESs is the presence of electroactive bacteria that form a biofilm on the anode electrode transforming organics into electrons, protons, smaller molecules and carbon dioxide. The electrons are transported to the anode electrode through direct or mediated electron transfer [15]. Electrons move to the external circuit and, in the case of a microbial fuel cell (MFC), generate electricity whilst an oxidant is reduced at the cathode [[9], [10], [11]]. In the case of a microbial electrolysis cell (MEC), a small external power source is applied to push the desired reaction forward with production of valuable products such as methane, alcohols, acetate, hydrogen [12,16,17]. A wide variety of wastewater types have been investigated successfully indicating that electroactive bacteria within BESs can be versatile for treating diverse pollution sources [18,19]. Diverse oxidants have also been used at the cathode with the preference of oxygen due to its high redox potential and natural availability at no cost and no weight [20,21].

Despite the idea of MFCs being quite promising, several challenges and drawbacks hinder their large-scale commercialisation [[22], [23], [24]]. However, numerous advancements have been achieved in understanding the electron transfer mechanisms within the anodic biofilm and in advancing the anode and cathode materials and their optimum design for energy/power harvesting [[9], [10], [11]] for practical applications. Particularly, the electron transfer mechanism occurs directly through membrane cytochromes or nanowires to the solid electrode or through catabolites or mediators [[25], [26], [27], [28]].

Anode materials have been significantly advanced in terms of increase in the interface bacteria/electrode for enhancing the electron transfer. Anode electrodes need to be electrically conductive, have mechanical strength and be resistant to pollutant-containing environments [[29], [30], [31], [32], [33]]. Moreover, the anode materials have to be low-cost in order to be scaled-up without influencing the overall cost. Carbonaceous-based materials and stainless steel have been identified as the most promising anode electrodes [9,10,[34], [35], [36]] even if recently these materials have been extended also to other metals [37,38].

Cathode electrodes have been significantly improved in terms of the air-breathing cathode structure and catalyst selection. Oxygen reduction reaction (ORR) occurring in neutral media has several limitations due to the sluggish kinetics occurring in neutral media where the concentrations of reactants such as H^+^ and OH^−^ is limited to 10^−7^ M [[39], [40], [41], [42]]. In order to accelerate the reaction, a catalyst is usually added into the cathode structure. These catalysts can be based on platinum group metals (PGMs) [[43], [44], [45]] or carbonaceous materials [[46], [47], [48]] or platinum group metal-free (PGM-free) with transition metals (e.g. Fe, Mn, Co and Ni) [[49], [50], [51], [52]]. Platinum-based catalysts are expensive and non-durable within wastewater environments due to pollutants that deactivate their catalytic centers [[53], [54], [55]]. A rapid decrease in activity has been reported with anions containing sulfur, chlorine and nitrogen [[53], [54], [55]]. Carbonaceous materials such as graphene [56,57], carbon nanotubes [58,59], modified carbon black [[60], [61], [62]], carbon nanofibers [63] and activated carbon [64,65] are largely used as cathode catalyst. Activated carbon seems to be the right choice since it is cost-effective, commercially available and durable over long terms operations [66,67]. In the past decade, PGM-free catalysts based on transition metals have also gained attention worldwide due to higher catalytic activity compared to both platinum and carbonaceous catalysts [68,69]. Large-scale production and relatively high costs still prevent its commercial implementation [54].

Hundreds of designs have been exploited with different shapes and volumes including cylindrical, rectangular, squared and flat shapes [[70], [71], [72], [73], [74]]. The volume of the reactors varies between microliters and hundreds of liters depending on the application and the purpose [[75], [76], [77], [78]]. The common point widely agreed is that in order to increase the power output and the organic removal, the surface to volume ratio has to be optimised in order to avoid “dead volume” in which antagonistic reactions such as fermentation and methanogenesis might occur without producing useful electricity [[75], [76], [77], [78]]. Generally, smaller reactors obtain higher power density due to the higher surface to volume ratio [[75], [76], [77], [78], [79]].

Due to the sluggish anodic and cathodic reactions in neutral media and also due to the low organics concentration and the relatively low operating temperatures, the power obtained from MFCs is quite low, which makes it difficult, but not impossible, to harvest for practical applications [10]. In order to improve the quality of the power output, MFCs are connected with external supercapacitors that are able to boost the current/power and deliver it under pulsed mode on demand [80,81]. A detailed review on the topic has recently been reported [82]. Diverse successful examples about deployment of practical applications have also been presented [[83], [84], [85], [86]]. Moreover, it was shown that intermittent MFC operations were able to increase the current/power output compared to the continuous operation [87,88].

In the past few years, supercapacitive biological fuel cells, both microbial [[89], [90], [91], [92], [93]] and enzymatic [[94], [95], [96], [97]], were also presented and widely studied. In the case of MFCs, the anode and the cathode electrodes of the MFCs were considered as the negative and positive electrodes of an internal supercapacitor. Galvanostatic discharges were presented and, due to redox reactions occurring on the two electrodes, the electrodes were also self-recharged. Following this line of work, in this study, a self-stratified MFC fed in continuous flow with urine was studied and considered like an internal supercapacitor. This type of membraneless MFC was developed in order to scale-up the technology with minimal performance losses [[98], [99], [100]]. With this particular MFC concept, it was shown before that self-stratifying MFCs could be scaled in width and length, from milliliter to liter scale, without decrease in power output and treatment efficiency [99,100]. Recently, the same design was shown to be scalable in height between 3.5 cm and 11.5 cm total MFC height [101]. This scalability relies in the fact that whilst the size of the reactor increases, the areal density of the electrode reactions remains constant. In other words the density of the reaction sites/surfaces is independent from the size, in the width and length dimensions, provided that the diffusion distances are kept minimal and constant. This type of MFC, developed for practical applications, has been taken out from laboratory trial [99] to field trial under real conditions of use [84].

Once the parameters of interest during the discharge were collected utilising anode and cathode materials of the MFC, equivalent series resistance (ESR) was then diminished and overall apparent capacitance was increased integrating supercapacitive features into the negative and the positive electrode by the use of an activated carbon layer. These variations allowed increasing the overall performance and time for full discharge significantly.

## 2 Materials and method

### 2.1 Electrodes materials

In the case of the control experiments, the anode (negative electrode) was composed by carbon veil (20 g m^−2^) with dimension of 2 × 5 cm that was wrapped several times into a rectangular shape and wrapped through a stainless steel wire. The projected area was 0.8 cm^2^ (0.8 × 1.0 cm) and corresponded to a weight of 0.020 g. The cathode (positive electrode) was instead composed by a mixture of activated carbon (AC, 800 m^2^ g^−1^, SK1 P75, CPL Carbon Link, UK) and polytetrafluoroethylene (PTFE) blended and pressed over a stainless steel mesh 316 (#18/0.45 mm ø wire). The thickness of the AC/PTFE material was roughly 1 mm (included the mesh), the projected area was 0.8 cm^2^ and the AC/PTFE mixture had a weight of 0.228 g. AC/PTFE loading was 0.101 ± 0.002 g cm^−2^. The control experiment was named **SC-MFC-control**.

In the case of capacitive negative electrode experiment, the SC was named as **SC-MFC-CapNE**, the carbon veil was then decorated adding a thin layer of AC/PTFE with a dimension of 2.5 × 2 cm by a thickness of 50 μm and weight 0.033 g, prepared separately. The thin film was then inserted into the carbon veil once it was wrapped and folded giving a total weight of 0.053 g.

In order to investigate the effect of the capacitive materials for the positive electrode, two identical air breathing cathodes (double geometric area compared to SC-MFC-control) were inserted into the electrochemical cell and the SC was named **SC-MFC-CapPE**.

### 2.2 Supercapacitor set up and operations

**Fig. 1:**
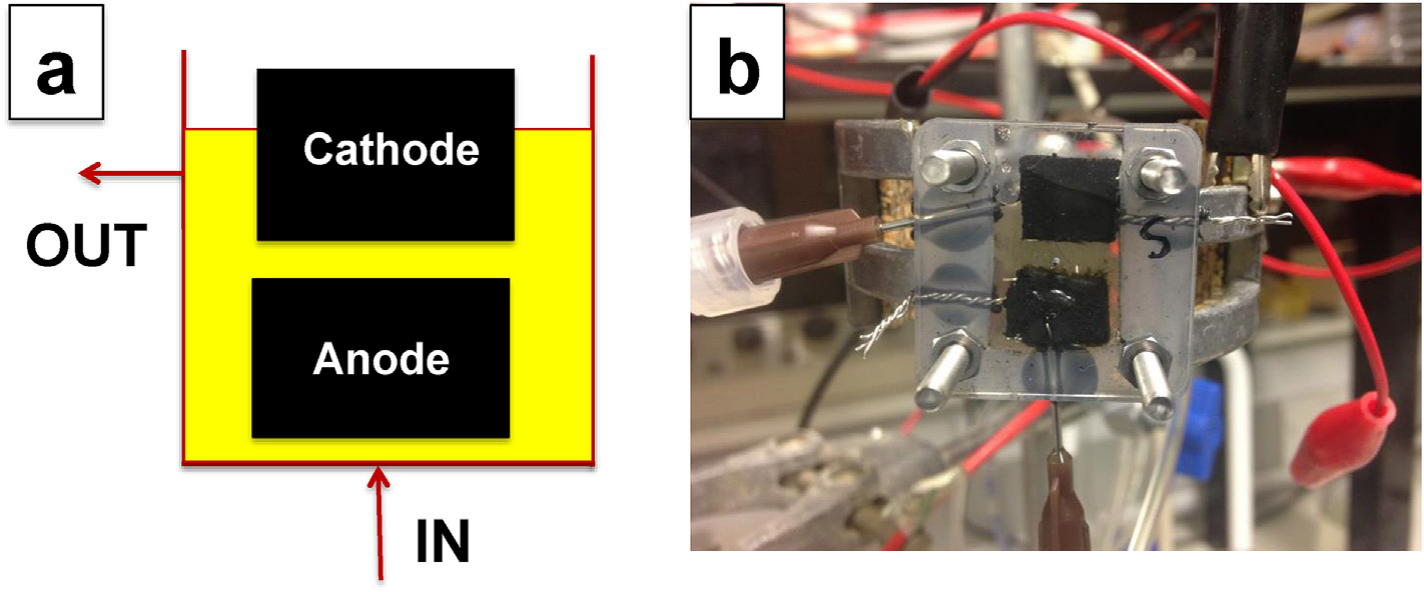
Schematic of the SC-MFC and an image of the SC-MFC.

A plastic cell with dimensions of 30 × 30 × 10 mm (height-length-depth) was used to accommodate the electrodes. The schematic and the image of the cell used during this investigation are showed in Fig. 1. The empty volume of the cell was 550 μL (including the electrodes). The urine displacement volume was 400 μL (including the electrodes) probably because 150 μL of electrolyte was retained within the porous electrodes. The cell featured a planar configuration: the negative electrode (NE) (anode of the MFC) was fixed in the lower part of the electrochemical cell while the positive electrode (PE) (cathode of the MFC) was instead fixed in the upper part of the cell (Fig. 1). No separator was used. The distance between the negative and the positive electrode was 1 mm.

The SC-MFC (supercapacitive MFC) operated with aqueous electrolyte that in this case was human urine collected from anonymous male donors. The electrolyte had relatively constant solution conductivity and pH over time that were 28.1 mS cm^−1^ and 9.2 respectively. The SC-MFC was operated in continuous flow as fresh solution without recirculation using a peristaltic pump (205CA cassette, Watson-Marlow Inc, UK) at a flow rate of 0.041 mL min^−1^ that remained constant during the entire operations. The liquid level was fully immersing the negative electrode and partially (roughly ¾) the positive electrode that was in part exposed to air (the remaining ¼). It was previously shown that this configuration was the best performing in SSM-MFCs [102]. After leaving the cell in open circuit, the electrodes were connected with an external resistor of 3 kΩ in order to stimulate the biofilm growing on the negative electrode like in the case of a microbial fuel cell. After the cell voltage was constant, the MFC was electrochemically studied like a supercapacitor (SC).

### 2.3 Electrochemical measurements

Galvanostatic discharges (GLV) were done using a Biologic SP-50 at different current pulses (**i**_**pulses**_) and different time (**t**_**pulse**_). A three electrode configuration was used for GLVs. The negative electrode was used as counter, the positive electrode as working and Ag/AgCl 3 M KCl was used as reference.

The cell was left in rest (**V**_**max,OC**_), the self-stratification within the cell was able to charge negatively and positively the two electrodes named as positive electrode (**PE**) and negative electrode (**NE**) without the support of external voltage. Then **i**_**pulse**_ was applied for a certain **t**_**pulse**_, the cell self-recharged itself going back to the initial value in rest conditions (**V**_**max,OC**_).

After the SC-MFC was left in rest, the GLV discharge was carried out at **i**_**pulse**_ and initially a vertical voltage drop (**ΔV**_**ohmic**_) can be observed. The ohmic losses of the SC-MFC system named as equivalent series resistance (**ESR**) that includes the ohmic resistance of the two electrodes and the electrolyte can be calculated according to eq. (1):

(1) $$ESR=\;\frac{\Delta V_{ohmic}}{i_{pulse}}$$

During the GLV discharges, the profiles of the single electrode were also recorded by inserting the reference electrode (RE) between **NE** and **PE**. The RE was inserted exactly in the middle of the chamber at equal distance between **NE** and **PE** in order to share equally the ohmic resistance of the electrolyte. Therefore eq. (1) can be rewritten as below:

(2) $$ESR=R_{NE}+R_{PE}$$

where **R**_**NE**_ and **R**_**PE**_ are the electrode resistances of the negative electrode and the positive electrode that also includes the ohmic term from the electrolyte. **R**_**NE**_ and **R**_**PE**_ can be calculated knowing the initial potential drop of the negative and positive electrode respectively as shown in eq. (3) and eq. (4):

(3) $$R_{NE}=\frac{\Delta V_{ohmic,NE}}{i_{pulse}}$$

(4) $$R_{PE}=\frac{\Delta V_{ohmic,PE}}{i_{pulse}}$$

After the vertical drop, the voltage reaches a new voltage point named as **V**_**max**_ and afterwards, the voltage continues to decrease during the electrostatic discharge of the self-polarised electrodes. The apparent capacitance of the SC-MFC (**C**_**SC**_) can be measured knowing the **i**_**pulse**_ and the variation of the voltage (excluding the initial vertical drop) named as **ΔV**_**capacitive**_ during the discharge time (**t**_**pulse**_) as shown in eq. (5):

(5) $$C_{SC}=\frac{i_{pulse}}{\frac{dV_{tot}}{dt}}=\frac{i_{pulse}}{s}$$

with ‘s’ indicating the slope of the voltage over time. In this specific case, not only electrostatic discharge is taking place but redox reactions occurring at the electrodes might contribute and different impact on the voltage trend over time depending on current rates. Therefore the term apparent capacitance describes better the mixed regime occurring in this case, that gives rise to the linear voltage decrease during galvanostatic pulses.

In order to increase **t**_**pulse**_ and the power/energy output, **ΔV**_**capacitive**_ should be minimized and in parallel **C**_**SC**_ should be maximized. The electrode potential profiles were measured over time by introducing the reference electrode. This enabled evaluation of the apparent capacitance of the single electrodes according to eq. (6) and eq. (7):

(6) $$C_{NE}=\frac{i_{pulse}}{\frac{dV_{NE}}{dt}}$$

(7) $$C_{PE}=\frac{i_{pulse}}{\frac{dV_{PE}}{dt}}$$

**C**_**SC**_ is related to the apparent capacitance of negative (**C**_**NE**_) and positive (**C**_**PE**_) electrodes through eq. (8):

(8) $$C_{SC}={\left(\frac{1}{C_{NE}}+\frac{1}{C_{PE}}\right)}^{-1}$$

Power and energy are very important parameters to consider when evaluating the electrochemical performance of the SC-MFC. The energy during a pulse, named as **E**_**pulse**_, can be calculated from the area below the voltage profile over time between the **V**_**max**_ and the final voltage point (**V**_**final, pulse**_) multiplied by **i**_**pulse**_ as shown in eq. (9):

(9) $$E_{pulse\;}=i_{pulse}\;\overset{t}{\underset{0}{\int }}V\;dt$$

The power produced during a pulse, named as **P**_**pulse**_, was consequently the ratio between **E**_**pulse**_ and the **t**_**pulse**_ as shown in eq. (10):

(10) $$P_{pulse}=\frac{E_{pulse}}{t_{pulse}}$$

In this current work, **t**_**pulse**_ of 0.01s, 0.1 s, 0.5 s, 1 s and 5 s were used for evaluating **P**_**pulse**_.

## 3 Results and discussion

### 3.1 Self-stratification and self-charging

Supercapacitors are usually fabricated with similar materials. The two identical electrodes of the supercapacitors are charged through the utilisation of an external power supply. Therefore, the external power supply allows the two electrodes to become positively and negatively charged. In the current case, both negative and positive electrodes are composed of carbonaceous materials as described in detail in Section 2.1. The difference to a traditional supercapacitor is the presence of bacteria within the liquid electrolyte and the electrodes arrangement (Fig. 1) that allow the generation of two regions within the electrolyte due to the self-stratification of the electrolyte [[100], [101], [102]]. In fact, the electrolyte is exposed to air on the upper part and oxygen is consumed going in depth until an anaerobic zone is created. Therefore, the bottom part of the cell, where the negative electrode is located, is completely anaerobic, while the upper part, where the positive electrode is located instead, is at least partly aerobic. The generation of these two different regions allows the selections of a diverse bacterial consortium within the lower and the upper parts. Once these two regions are created, the negative electrode is negatively polarised and the positive electrode is positively polarised. On the negative electrode, the electroactive biofilm colonises the electrode and is capable of oxidising organics while on the positive electrode, the biofilm composed of bacteria tolerant to oxygen or alternative oxidised ions (e.g. nitrate, sulfate, etc) is capable of carrying out the reduction reaction. The same principle was shown previously in self-stratifying membraneless microbial fuel cells (SSM-MFC) where similar systems were able to run in continuous flow, fed with human urine under a constant load [100,102]. Once fresh food was provided and oxygen was present, the SSM-MFC was capable of running continuously for over 30 days [102]. In these two regions, different microorganisms are present and diverse redox potential levels are also created.

Therefore, if the supercapacitive features of the two electrodes are considered, the SC-MFC is self-powered due to the two redox reactions occurring at the two electrodes. In this work, the electrodes of the system are considered as electrodes of an internal supercapacitor that is self powered through the two redox reactions occurring at the two electrodes and is capable of storing electrostatic charges from the electrolyte and discharge them with high and fast current pulses. The negative electrode (on the bottom of the SC-MFC) is negatively charged and therefore it will electrostatically attract the opposite charges (positive) from the electrolyte. In parallel, the positive electrode is positively charged attracting negative charges from the surrounding solution. During the galvanostatic discharges, the counter-ions are released into the electrolyte. Once the discharge is completed, the electrodes are self-polarised due to their redox reactions occurring on the electrode and therefore will attract back the counter-ions from the electrolyte. Theoretically, if the two redox reactions self-polarise the electrodes, the discharge/self-recharge process could occur indefinitely, as long as fuel/substrate is provided to the microflora thriving in the system.

### 3.2 Characterisation of the control supercapacitor

**Fig. 2:**
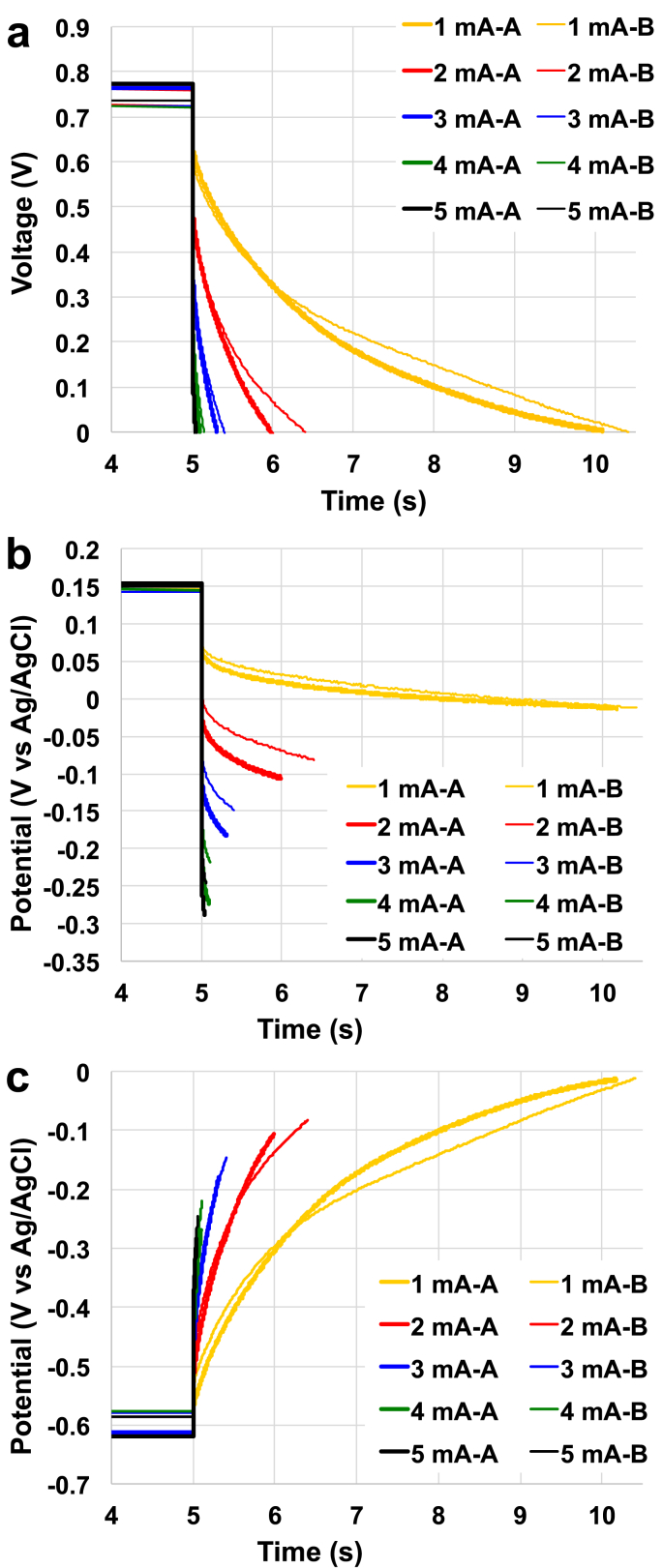
Overall (a), positive electrode (b) and negative electrode (c) complete discharges for the SC-MFC-control at different current pulses.

The SC-MFC-control fabricated with carbon veil as negative electrode and AC/PTFE pressed over a SS mesh as positive electrode was left in rest and **V**_**max,OC**_ was recorded. NE had a measured potential value of −599 ± 19 mV (vs Ag/AgCl) and PE had a potential value of +150 ± 3 mV (vs Ag/AgCl). Those values can be explained by the two red-ox reactions occurring on the anode and a cathode of an MFC operating with urine as previously shown [102]. **V**_**max,OC**_ of the control supercapacitor was 749 ± 22 mV. Complete galvanostatic discharges were then carried out at different **i**_**pulses**_ and the voltage profile of the supercapacitor (Fig. 2a) and the single electrode (Fig. 2b and 2.c) are presented. **ESR** and apparent capacitance can be extrapolated from these measurements. ESR was calculated to be 63 ± 4 Ω and the contribution of the NE and PE was 20 ± 3 Ω and 43 ± 2 Ω, respectively. NE corresponded to the 30% of the ESR while PE was the 70% of the ESR. The lower the **i**_**pulse,**_ the higher was the time of complete discharge (**t**_**discharge**_). Complete discharge occurred after 5.30 ± 0.12 s, 1.20 ± 0.20 s, 0.36 ± 0.05 s, 0.13 ± 0.03 s and 0.05 ± 0.01 s for **i**_**pulse**_ of 1 mA, 2 mA, 3 mA, 4 mA and 5 mA respectively.

Apparent capacitance of the overall SC-MFC-control (**C**_**SC**_) increased with lower **i**_**pulse**_. In fact **C**_**SC**_ increased from 0.84 ± 0.16 mF (**i**_**pulse**_ of 5 mA) to 8.20 ± 0.54 mF (**i**_**pulse**_ of 1 mA). Once again, the single electrode profiles helped to separate the single contribution. **C**_**NE**_ measured 9.66 ± 0.67 mF, 5.49 ± 1.05 mF, 3.10 ± 0.41 mF, 1.99 ± 0.35 mF and 1.30 ± 0.22 mF at **i**_**pulse**_ of 1 mA, 2 mA, 3 mA, 4 mA and 5 mA, respectively. In parallel, **C**_**PE**_ measured 54.2 ± 2.9 mF, 21.1 ± 5.7 mF, 8.9 ± 2.2 mF, 4.1 ± 1.6 mF and 2.3 ± 0.6 mF at **i**_**pulse**_ of 1 mA, 2 mA, 3 mA, 4 mA and 5 mA, respectively. These values indicated that the apparent capacitance of the negative electrode is much lower compared to **C**_**PE**_, in turn affecting the **C**_**SC**_. The complete discharge curves indicated that PE had higher ohmic resistance but also higher apparent capacitance. The fact that pulses were short was mainly due to the poor apparent capacitance of the NE.

### 3.3 Strategy for improving anode apparent capacitance

**Fig. 3:**
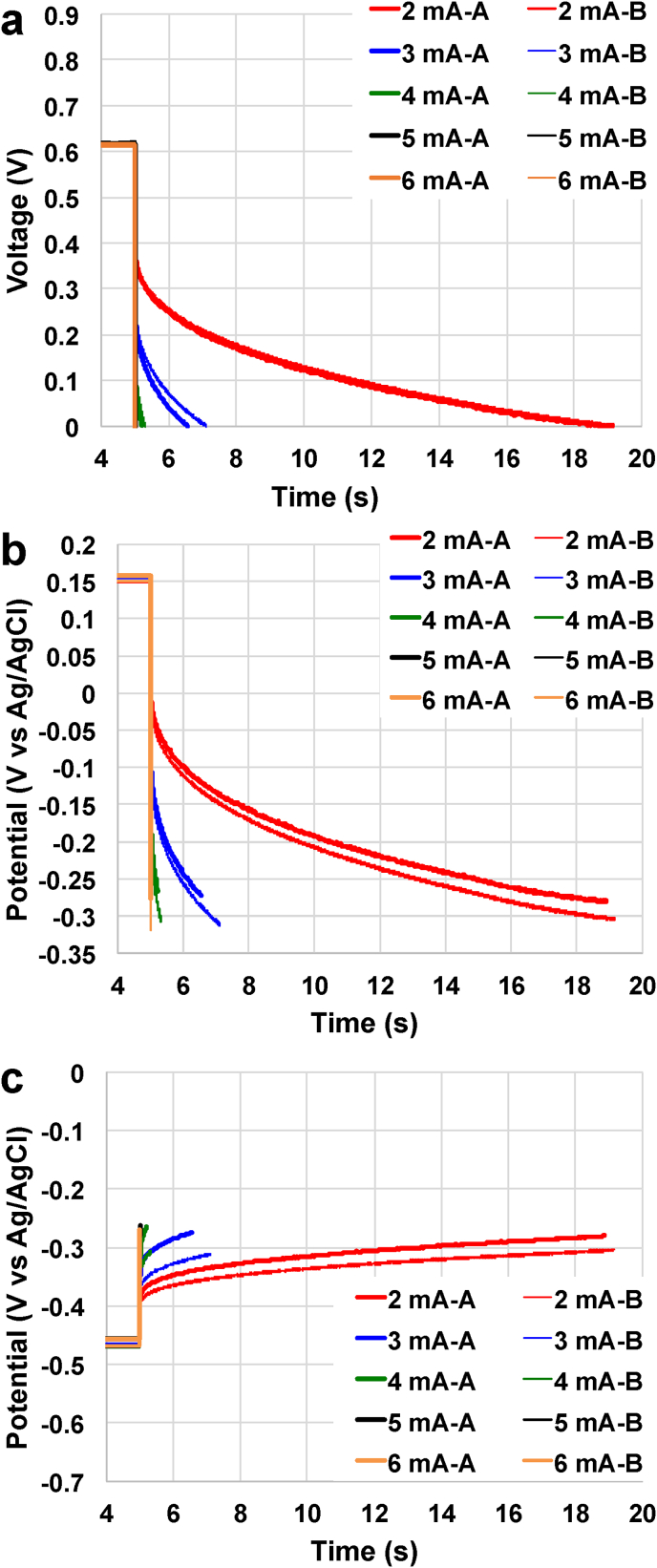
Overall (a), positive electrode (b) and negative electrode (c) complete discharges for the SC-MFC-CapNE with improved anode at different current pulses.

Despite the NE having a lower ohmic resistance compared to the PE, the very low apparent capacitance of the NE allowed for only short discharges to be achieved. Therefore, a capacitive electrode composed by AC/PTFE pressed over a SS mesh (described in section 2.1) was embedded within the existing carbon veil wrapped electrode with the intention of increasing the NE apparent capacitance and elongating the **t**_**pulse**_ for the overall discharge. The complete discharges at different **i**_**pulse**_ for the SC-MFC-CapNE with improved anode were carried out and the overall profile (Fig. 3a) as well as the profiles of the single electrode (Fig. 3b and 3.c) are presented. Overall and single electrode complete discharge with **i**_**pulse**_ of 1 mA was reported in the Supporting Info (Fig. S1).

Compared to the SC-control, in rest condition, NE had a higher potential value of −468 ± 2 mV (vs Ag/AgCl). PE had similar potential measuring +153 ± 5 mV (vs Ag/AgCl). This means that the reduction in NE potential was roughly 150 mV corresponding also to a reduction in the **V**_**max,OC**_ of the SC-MFC-CapNE with capacitive anode that was 620 ± 6 mV. The change in NE potential might be due to the presence of PTFE, which is the hydrophobic agent used for binding the AC particles to the SS mesh. Hydrophobic materials such as PTFE are well know to discourage bacterial attachment on the electrode and therefore probably less bacterial biomass was attached to the electrode and the anaerobic zone was difficult to establish as previously shown [62,[103], [104], [105]].

**ESR** was evaluated through eq. (1) and was quantified as 58 ± 2 Ω, which was slightly lower but similar to the **ESR** of the SC-MFC-control. NE had an ohmic resistance of 17 ± 1 Ω while PE had the **R**_**PE**_ of 41 ± 1 Ω. The main goal of the addition of the capacitive electrode within the negative electrode was to enhance the apparent capacitance of the electrode and therefore of the overall SC-MFC. Once again, the overall apparent capacitance increased with the decrease of the **i**_**pulse**_. Among the range of **i**_**pulse**_ investigated (between 1 mA and 6 mA), **C**_**SC**_ varied between 0.09 ± 0.00 mF (**i**_**pulse**_ of 6 mA) to 283.0 ± 7.7 mF (**i**_**pulse**_ of 1 mA). The recording of each electrode separately allowed estimation of the negative and positive electrode apparent capacitance. **C**_**NE**_ had an apparent capacitance of 870 ± 52 mF, 240 ± 18 mF, 59 ± 13 mF, 13.5 ± 3.4 mF, 1.82 ± 0.50 mF, and 0.33 ± 0.07 mF at **i**_**pulse**_ of 1 mA, 2 mA, 3 mA, 4 mA, 5 mA and 6 mF, respectively. In parallel, at the same **i**_**pulse**_, **C**_**PE**_ measured 420.4 ± 4.2 mF, 87.3 ± 0.6 mF, 21.5 ± 1.9 mF, 5.7 ± 0.8 mF, 0.83 ± 0.07 mF and 0.12 ± 0.01 mF. It can be concluded that the strategy of adding a capacitive electrode within the NE was successful allowing the substantial increase in the **C**_**NE**_ that became higher compared to **C**_**PE**_. As a consequence of the increase in overall **C**_**SC**_, and despite similar **ESR**, the time of total discharge increased substantially; in fact the discharges were completed after 148.1 ± 4.1 s, 14.1 ± 0.1 s, 1.85 ± 0.27 s, 0.27 ± 0.05 s, 0.021 ± 0.003 s and 0.0014 ± 0.0001 s for **i**_**pulse**_ of 1 mA, 2 mA, 3 mA, 4 mA, 5 mA and 6 mA, respectively.

### 3.4 Strategy for lowering cathode ESR

**Fig. 4:**
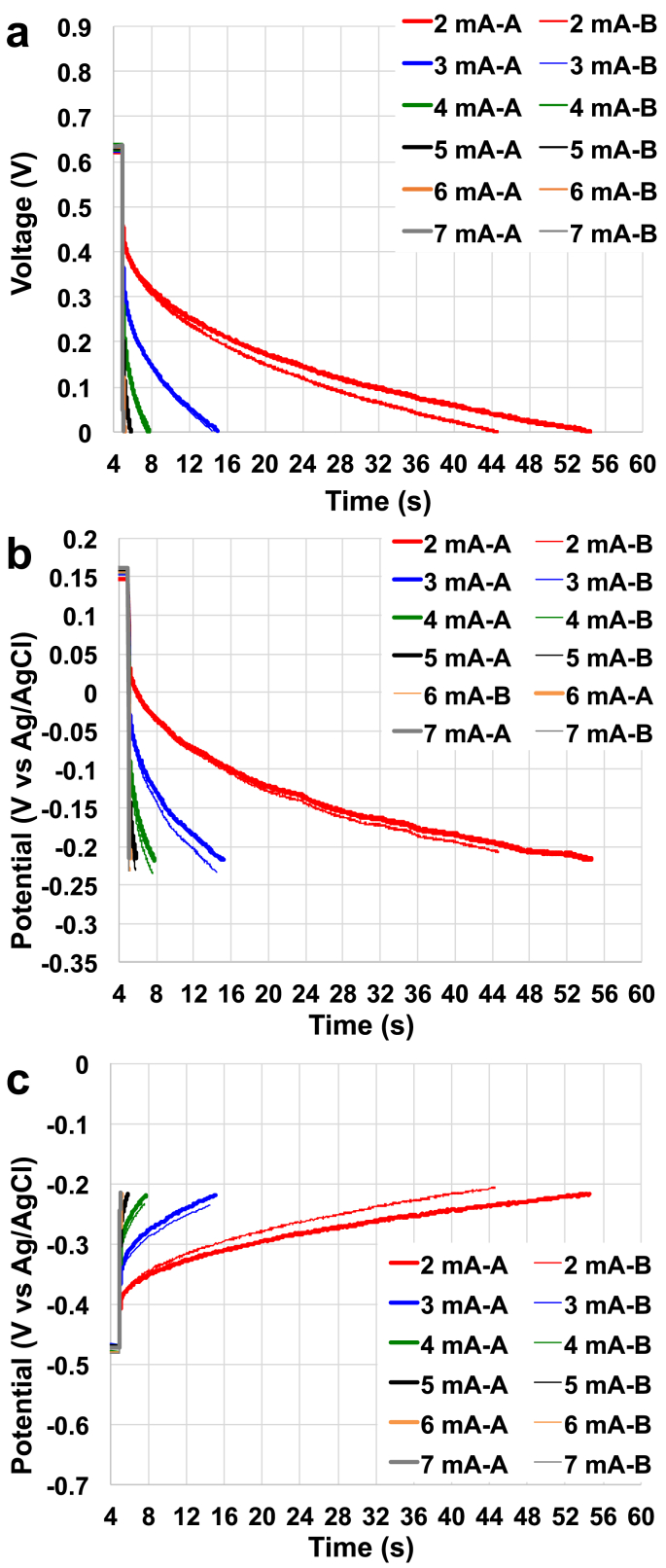
Overall (a), positive electrode (b) and negative electrode (c) complete discharges for the SC with improved anode and double cathode at different current pulses.

The increase in apparent capacitance within the negative electrode enhanced the performance with longer time necessary for discharging completely the SC-MFC. The positive electrode still suffered from high **ESR** and after the actuation of the first strategy, the **C**_**PE**_ was lower compared to **C**_**NE**_. Therefore, a second capacitive electrode was added into the plastic cell in contact with the PE. Complete galvanostatic discharges of the SC-MFC-CapPE were then carried out and overall (Fig. 4a) and single electrode (Fig. 4b and.c) profiles were recorded. Full discharge (overall and single electrode) for **i**_**pulse**_ of 1 mA is shown in the Supporting Information (Fig. S2). **V**_**max,OC**_ and the potentials of the single electrodes remained constant compared to the previous case of study measuring 629 ± 6 mV, −471 ± 2 mV (vs Ag/AgCl) (NE) and +156 ± 5 mV (vs Ag/AgCl) (PE), respectively. With the addition of the second PE, the ESR dropped to ± 48 ± 1 Ω. This value was ≈10 Ω lower compared to the previous supercapacitors. While **R**_**NE**_ remained practically constant (16 ± 1 Ω), the contribution of the positive electrode (**R**_**PE**_) measured 32 ± 1 Ω with a reduction of 25% compared to the control SC. Despite the decrease, **R**_**PE**_ corresponded to the 65% of the **ESR** and **R**_**NE**_ contributed for 35% of **ESR**. Hence, also the SC-MFC-CapPE apparent capacitance increased substantially varying from 1.64 ± 0.22 mF (**i**_**pulse**_ 7 mA) to 551 ± 36 mF (**i**_**pulse**_ 1 mA). As can be seen from Fig. 4.a, the decrease in potential after the ohmic drop is similar for both **NE** and **CE** indicating similar apparent capacitance. Time for the total discharge increased even more and was measured to be 307 ± 19 s, 45.3 ± 4.3 s, 9.91 ± 0.33 s, 2.71 ± 0.12 s, 0.86 ± 0.01 s, 0.19 ± 0.02 s and 0.04 ± 0.01 s for **i**_**pulse**_ of 1 mA, 2 mA, 3 mA, 4 mA, 5 mA, 6 mA and 7 mA, respectively.

**Fig. 5:**
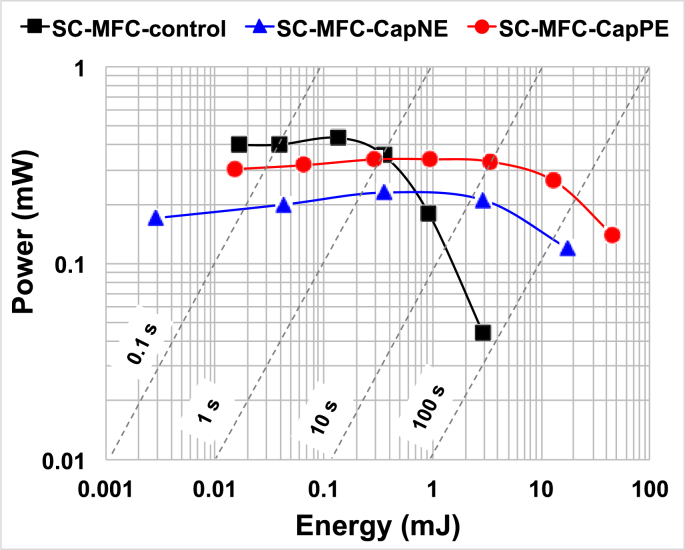
Ragone plots of SC-MFC-control, SC-MFC-CapNE and SC-MFC-CapPE. Dash lines indicate characteristic discharge time.

The Ragone plots reported in Fig. 5 compared the energy and power performance of the three cells studied at different current densities corresponding to different discharge times. The plots show that for fast discharges the SC-MFC-control is the best performing cell followed by SC-MFC-CapPE and SC-MFC-CapNE. For discharges lasting more than 5 s, the trend changes and the addition of capacitive features into negative and positive electrodes is beneficial with the SC-MFC-CapPE outperforming the other SC-MFCs. These trends can be explained considering **V**_**max**_, **ESR** and C_SC_. Indeed, at shorter times, **V**_**max**_ is the key parameter that mainly affects power delivered and SC-MFC-control is the cell with the highest initial voltage. Instead, in the case of longer discharge time, given that capacitance shapes the voltage trend over time, power delivered is largely and mainly affected by the apparent capacitance rather than **V**_**max**_ therefore SC-MFC-CapNE and SC-MFC-CapPE outperform the control. SC-MFC-CapPE had higher power output compared to SC-MFC-CapNE due to its lower ESR.

### 3.5 Power curves

**Fig. 6:**
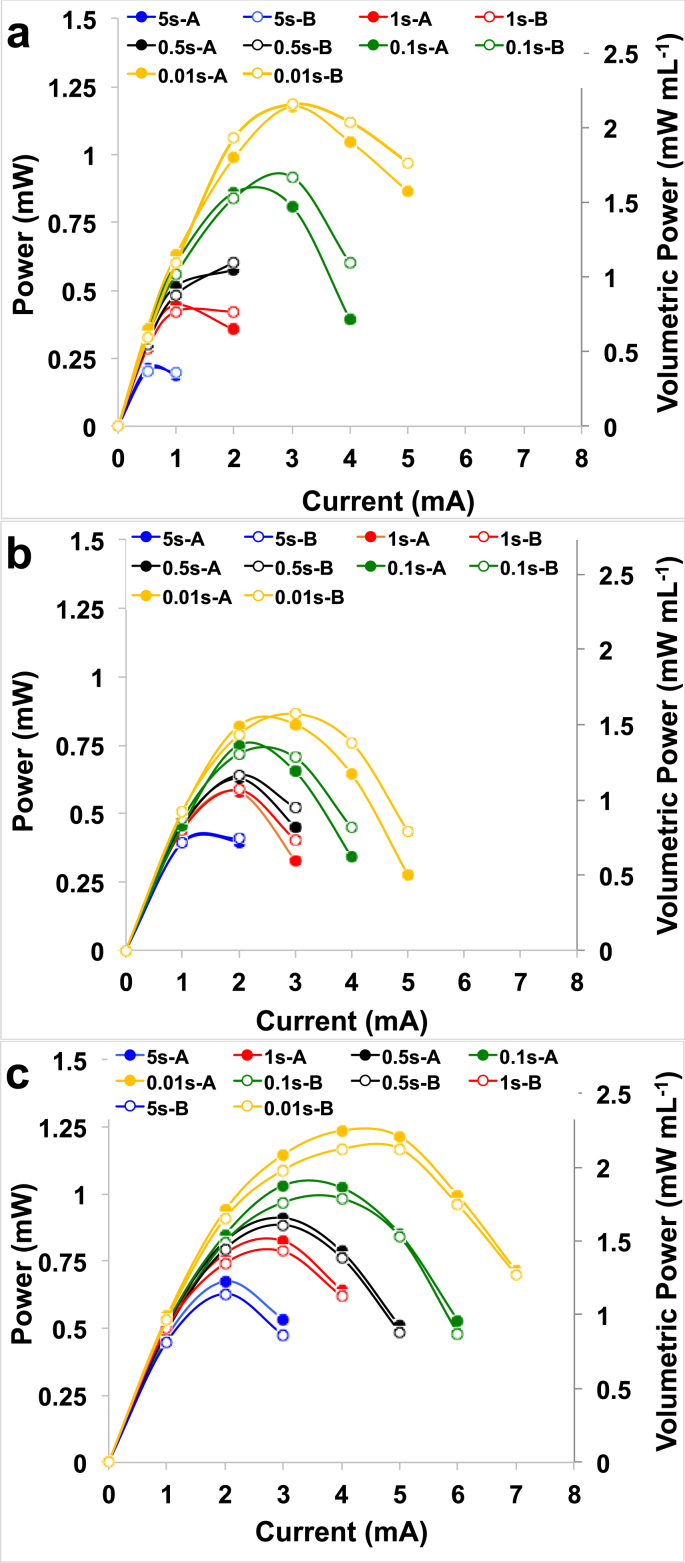
P_pulse_ for SC-MFC-control (a), SC-MFC-CapNE (b) and SC-MFC-CapPE (c) at t_pulse_ of 0.01 s, 0.1 s, 0.5 s, 1 s and 5 s.

Another important parameter to consider is the power produced by the supercapacitors at different **t**_**pulse**_ previously described in eq. (10). The **t**_**pulse**_ considered were 0.01 s, 0.1 s, 0.5 s, 1 s and 5 s. The power curves obtained by the galvanostatic discharges are presented in Fig. 6. The maximum value of **P**_**pulse**_ for each **t**_**pulse**_ is presented in Table 1. As expected, the larger **t**_**pulse**_, the lower was the power produced due to the voltage decrease over time during the discharge. Considering the three different SC-MFCs, at long **t**_**pulse**_ (5 s), the maximum power obtained by the SC-MFC-control was 0.21 ± 0.01 mW (0.38 ± 0.02 mW ml^−1^) and it was lower than the one produced by SC-MFC-CapNE (0.40 ± 0.01 mW or 0.73 ± 0.01 mW ml^−1^) and the one produced by SC-MFC-CapPE (0.65 ± 0.02 mW or 1.18 ± 0.04 mW ml^−1^). At shorter **t**_**pulse**_ (0.5 s), the maximum power recorded by the SC-MFC-control was 0.59 ± 0.01 mW (1.07 ± 0.03 mW ml^−1^) while SC-MFC-CapNE had a peak of power of 0.63 ± 0.01 mW (1.15 ± 0.01 mW ml^−1^) and SC-MFC-CapPE had the higher value of power peak (0.89 ± 0.01 mW or 1.63 ± 0.02 mW ml^−1^). Interestingly, at the shortest investigated **t**_**pulse**_ of 0.01 s, SC-MFC-CapPE had the higher peak of power produced of 1.20 ± 0.04 mW (2.19 ± 0.06 mW ml^−1^) that was also the highest value recorded in this current work. SC-control had a slightly lower peak power of 1.18 ± 0.01 mW (2.15 ± 0.01 mW ml^−1^) and SC-MFC-CapNE had the lowest power produced of 0.85 ± 0.02 mW (1.53 ± 0.04 mW ml^−1^) for the t_pulse_ of 0.01 s.

**Table 1:** Maximum values of P_pulse_ for different t_pulse_ represented in terms of overall power produced (mW) and volumetric power (mW ml^−1^).

Time	SC-MFC-control	SC-MFC-CapNE	SC-MFC-CapPE	SC-MFC-control	SC-MFC-CapNE	SC-MFC-CapPE
s	mW	mW	mW	mW ml^−1^	mW ml^−1^	mW ml^−1^
5	0.21 ± 0.01	0.40 ± 0.01	0.65 ± 0.02	0.38 ± 0.01	0.73 ± 0.01	1.18 ± 0.04
1	0.44 ± 0.01	0.58 ± 0.01	0.81 ± 0.02	0.79 ± 0.02	1.05 ± 0.01	1.47 ± 0.04
0.5	0.59 ± 0.01	0.63 ± 0.01	0.90 ± 0.01	1.07 ± 0.03	1.15 ± 0.01	1.63 ± 0.02
0.1	0.89 ± 0.03	0.73 ± 0.02	1.00 ± 0.02	1.61 ± 0.05	1.33 ± 0.03	1.83 ± 0.04
0.01	1.20 ± 0.03	0.85 ± 0.02	1.19 ± 0.05	2.19 ± 0.06	1.54 ± 0.03	2.16 ± 0.08

The addition of a capacitive negative electrode in the SC-MFC-CapNE led to a decrease in the **V**_**max,OC**_ of roughly 150 mV despite the **ESR** being similar. The results indicated that for short-term pulse (≤0.1 s), the addition of a capacitive negative electrode did not produce any advantage; in fact SC-MFC-control outperformed SC-MFC-CapNE. The addition of the capacitive positive electrode in the SC-MFC-CapPE still led to a decrease of ≈150 mV in the **V**_**max,OC**_ but also a reduction of 25% of the **ESR**. The decrease in **ESR** allowed for a slight increase in the power output compared to SC-MFC-control also for short pulses. For longer pulses (≥1 s), the improved apparent capacitance with the addition of capacitive negative and positive electrodes within the SC-MFC, influences substantially the power output despite lower **V**_**max,OC**_.

### 3.6 Long term operations

**Fig. 7:**
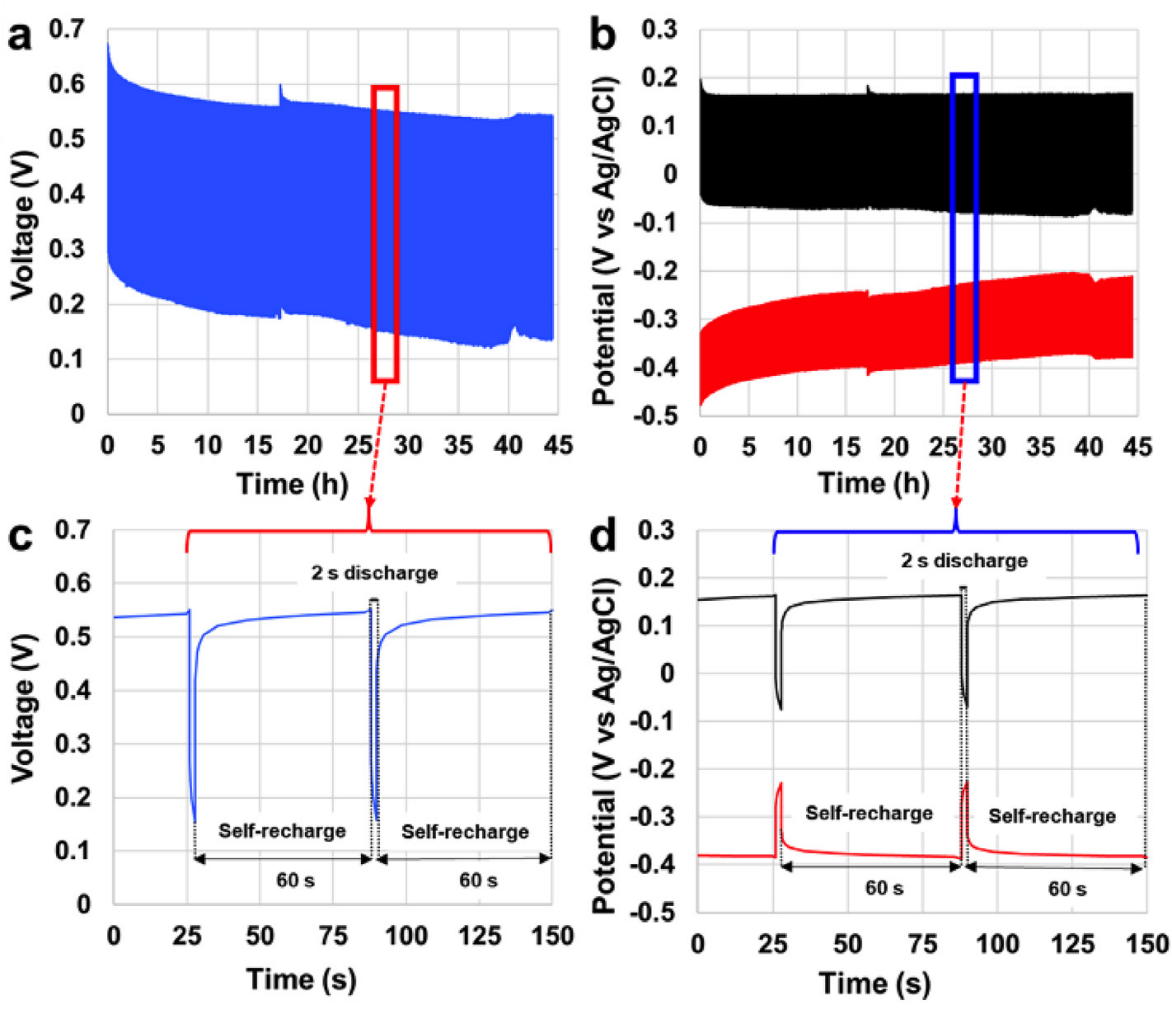
2600 cycles of discharges (2 s) and self-recharge (60 s) of SC-MFC-CapPE. Overall cell discharge (a) and single electrode discharge (b). Zoom of overall cell discharge (c) and single electrode discharge (d).

SC-MFC-CapPE was then discharged and self-recharged for roughly 2 days (44 h) that was equivalent to ≈2600 discharge/recharge cycles. Overall and single electrode profiles are represented in Fig. 7.a and Fig. 7.b respectively. The discharge had a **t**_**pulse**_ of 2 s and **i**_**pulse**_ of 2 mA. The time of self-recharge selected was 60 s. A close-up of two discharge and self-recharge cycles is shown after ≈27 h operations in Fig. 7.c and Fig. 7.d.

**Fig. 8:**
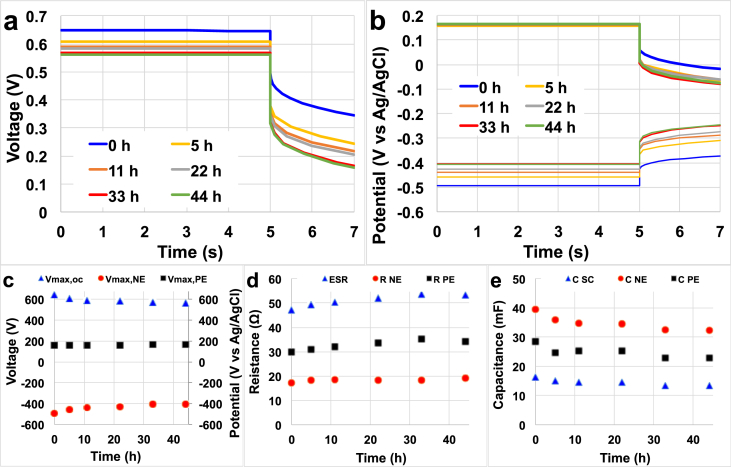
5 s rest and 2 s discharge at 2 mA after 0, 5, 11, 22, 33 and 44 h. Overall cell voltage (a) and single electrode potential (b) profiles. V_max_ (c), resistance (d) and apparent capacitance (e) trend over long terms operations.

In order to better understand the trend, the initial discharge and the discharges after 5 h, 11 h, 22 h, 33 h and 44 h were plotted separately and analysed. The overall discharges are presented in Fig. 8.a and the profiles of the **NE** and **PE** are presented in Fig. 8.b. The trend of parameters of interest such as V_max_, resistance and apparent capacitance during the 44 h operations are presented separately in Fig. 8.c, Fig. 8.d and Fig. 8.e respectively. **V**_**max,OC**_ tends to decrease over time measuring 647 mV initially, 608 mV after 5 h, 589 mV after 11 h and then the decrease continued slowly until a **V**_**max,OC**_ of 561 mV after 44 h (Fig. 8c). **V**_**max,PE**_ remained stable over the 44 h experiment measuring between +160 and + 165 mV (vs Ag/AgCl) (Fig. 8c). Furthermore, **V**_**max,NE**_ showed a continuous decrease in potential value starting from −494 mV (vs Ag/AgCl) initially, −458 mV (vs Ag/AgCl) at 5 h, −439 mV (vs Ag/AgCl) at 11 h, −428 mV (vs Ag/AgCl) at 22 h and it stabilised around −407 mV (vs Ag/AgCl) after 33 h and 44 h (Fig. 8c). As the experiments were conducted in continuous flow, lack of fuel was not an explanation for the decrease in the NE potential. The only possible explanation is the fact that 60 s time for self-recharge was sufficient for the PE to recover its initial **V**_**max,PE**_ but was not long enough for the NE to restore its initial potential. ESR increased over time until 22 h followed by a plateau (Fig. 8d). In particular, the initial **ESR** was 47 Ω and increased to 49 Ω and 50.5 Ω after 5 h and 11 h. At 22 h, the ESR stabilised at ≈53 Ω (Fig. 8d). **R**_**NE**_ initially was 17 Ω but after 5 h stabilised to ≈19 Ω. **R**_**PE**_ was initially 30 Ω and increased steadily up to 35 Ω at the end of the experiments (Fig. 8d). In addition, the **C**_**SC**_ decreased over time with initial values of 16.5 mF, 14.6 mF after 22 h and 13.5 mF at the end of the operation (Fig. 8e). It must be noted that the apparent capacitance values here presented are lower compared to the apparent capacitance calculated from the full discharges presented in Section 3.4. **C**_**NE**_ was higher than **C**_**PE**_ as can be seen in Fig. 8.b. **C**_**NE**_ decreased from the initial value of 39.6 mF (0 h) to 34.5 mF in the middle of the experiment (22 h) and 32.3 mF after 44 h (Fig. 8e). **C**_**PE**_ also decreased, varying from 28.6 mF initially to 23.0 mF after 44 h (Fig. 8e).

### 3.7 Outlook and future developments

In this work, a self-stratified microbial fuel cell fed with urine and operating in supercapacitive mode has been fabricated and investigated for the first time. The electrolyte volume was 0.55 mL. The specific design showed in Fig. 1 allowed developing self-stratification within the chamber with the lower part consuming oxygen and generating an anaerobic zone while the upper part exposed to air generated an aerobic zone. The two regions within the chamber create a reducing environment – degrading organics under anaerobic conditions - on the bottom and an oxidising environment – reacting with oxygen - on the top [99]. The two carbonaceous electrodes were then self-charged without the utilisation of an external power source. The oppositely charged electrodes, were then able to attract counter-ions from the electrolyte composed of human urine, therefore operating as supercapacitor electrodes. Human urine is rich in dissolved salts as previously identified and has been shown to be an excellent fuel for BES [[106], [107], [108]]. The electrodes were then discharged at high current pulses. Once the discharge was completed, the two red-ox reactions at the two electrodes helped restore the electrochemical double layer on each electrode. A control SC-MFC was tested using the electrode materials previously identified for SSM-MFC [100]. The tests showed high **V**_**max,OC**_ but very low apparent capacitance especially due to the poor capacitive features of carbon veil. The apparent capacitance of the negative electrode was then increased with the addition of a capacitive electrode incorporated into the electrode itself. This strategy allowed for increasing the apparent capacitance and the **t**_**pulse**_ of discharge but negatively affected the **V**_**max,OC**_ that decreased by 150 mV. Once the negative electrode apparent capacitance was enhanced, the ohmic resistance of the positive electrode still accounted for 70% of the total **ESR**, moreover, after the application of the first strategy, **C**_**PE**_ was lower than the **C**_**NE**_. A second strategy was then pursued to overcome these limitations and a second positive electrode, identical to the first one, was introduced in the chamber. **ESR** was diminished and **C**_**SC**_ was further increased. These two strategies allowed increasing the **t**_**pulse**_ during complete discharge and increasing substantially the power produced especially for long **t**_**pulse**_ periods. Durability tests were also conducted on the most performing SC-MFC during a period of time of 44 h in which ≈2600 discharge/recharge cycles were recorded. These cycles consisted of a discharge with 2 mA **i**_**pulse**_ at **t**_**pulse**_ of 2 s followed by a self-recharge period of 60 s. The results showed a decrease in the overall electrochemical performance mainly due to the negative electrode behaviour. In fact, while 60 s recharge time was enough for the positive electrode to recover its initial potential, this time was not sufficient for the negative electrode, which continued to progressively increase its potential. A more careful decision of recharge time should be selected for practical applications.

This work showed the possibility of creating a supercapacitor by using the self-stratification of the environment that self-charges the electrodes. Self-powered supercapacitors operating in municipal wastewater were previously presented successfully [[89], [90], [91], [92], [93],^109^]. In these previous examples, the power density achieved in supercapacitive microbial fuel cells was in the range of 0.1–0.5 mW mL^−1^ [[88], [89], [90], [91], [92],^107^] while in the present work the peak of power was above 1 mW mL^−1^. It was shown before that intermittent mode operation increases the power output [86,87,110]. Continuous mode operation allowed for a performance of the order of 0.01–0.1 mWmL^−1^ [111], which is one order of magnitude lower, compared to the intermittent mode operation. Also concerning the utilisation of human urine in MFCs, the pulsed power output was much higher compared to the previously reported examples [[98], [99], [100], [101], [102]].

This specific work indicates the possibility of SCs operating in other types of wastewater. To the best of the authors’ knowledge, this is the first self-powered supercapacitor operating with human urine. In contrast with municipal wastewater, human urine is much more conductive and has higher concentration of dissolved ions with solution conductivity measured above 20 mS cm^−1^. In fact, low electrolyte conductivity is often an enormous limitation in bioelectrochemical systems [112,113], therefore urine seems to alleviate this occurring problem. Once the limitation of the systems is identified, strategies dedicated to overcome the problems were undertaken to increase the performance. The best performing SC-MFC had a power pulse of 1.20 ± 0.04 mW or 2.19 ± 0.06 mW mL^−1^ at 0.01 s and 0.65 ± 0.02 mW or 1.18 ± 0.04 mW mL^−1^ at 5 s. This single SC seems to be limited mainly by its own potential difference that was below 0.75 V. For practical applications, a number of SC should be connected in series to boost the voltage to a level that can be practically utilised. Further investigation should consider the implementation of this self-powered supercapacitor for other wastewaters, other reductive/oxidative environments and into scaled-up system.

## 4 Conclusions

In this work, a self-powered supercapacitive microbial fuel cell (SC-MFC) operating with human urine was investigated. The redox reaction occurring on both electrodes allowed for self-charging of the electrodes. Electrochemical double layer was then formed on each electrode and counter-ions were attracted. Galvanostatic discharges were conducted at different current pulses. The control SC-MFC suffered from high positive electrode resistance and low apparent capacitance of the negative electrode. Supercapacitive features were improved adding activated carbon materials and ESR was reduced. The higher power pulses achieved were 1.20 ± 0.04 mW (2.19 ± 0.06 mW mL^−1^) at 0.01 s and 0.65 ± 0.02 mW (1.18 ± 0.04 mW mL^−1^) at 5 s time pulses. Long-term tests were conducted with discharges at 2 mA for 2 s and 60 s self-recharge times. ESR tended to increase over time while the apparent capacitance tended to decrease. The main losses were related to the negative electrode potential not able to fully recover its initial potential. For optimisation purposes, the discharge parameters should be tailored to specific application requirements, resulting in better performance.
