# Supplementary material for: Self-stratified and self-powered micro-supercapacitor integrated into a microbial fuel cell operating in human urine
Source: Electrochim Acta. 2019 Jun 1;307:241–52. doi: 10.1016/j.electacta.2019.03.194 (PMC6559283; doi:10.1016/j.electacta.2019.03.194)
Supplement: Multimedia component 1 [file mmc1.docx]

**Supporting information**

**Self-stratified and self-powered micro-supercapacitor integrated into a microbial fuel cell operating in human urine**

Carlo Santoro^1,*^, Alexis Walter^1^, Francesca Soavi^2^, John Greenman^3^, Ioannis Ieropoulos^1,3,**^

^1^ Bristol BioEnergy Centre, Bristol Robotics Laboratory, T-Block, UWE, Coldharbour Lane, Bristol BS16 1QY, UK

^2^ Department of Chemistry “Giacomo Ciamician”, Alma Mater Studiorum – Università di Bologna, Via Selmi, 2, 40126 Bologna, Italy

^3^ Biological, Biomedical and Analytical Sciences, UWE, Coldharbour Lane, Bristol BS16 1QY, UK

*corresponding author:

* Carlo Santoro. [carlo.santoro@uwe.ac.uk](mailto:carlo.santoro@uwe.ac.uk). [carlo.santoro830@gmail.com](mailto:carlo.santoro830@gmail.com)

** Ioannis Ieropoulos. ioannis2.Ieropoulos@uwe.ac.uk

**Figure S1. Overall (a), negative electrode (b), positive electrode (c) complete discharges for the SC-MFC-CapNE at i_pulse_ 1 mA**

**Figure S2. Overall (a), negative electrode (b), positive electrode (c) complete discharges for the SC-MFC-CapPE at i_pulse_ 1 mA**
